# Autoantibody Profiling and Anti-Kinesin Reactivity in ANCA-Associated Vasculitis

**DOI:** 10.3390/ijms242015341

**Published:** 2023-10-19

**Authors:** Federica Mescia, Shaghayegh Bayati, Elisabeth Brouwer, Peter Heeringa, Erik J. M. Toonen, Marijke Beenes, Miriam J. Ball, Andrew J. Rees, Renate Kain, Paul A. Lyons, Peter Nilsson, Elisa Pin

**Affiliations:** 1 Department of Medicine, University of Cambridge, Cambridge CB2 0SP, UK; federica.mescia@unibs.it (F.M.); pal34@cam.ac.uk (P.A.L.); 2 Cambridge Institute of Therapeutic Immunology and Infectious Disease, Jeffrey Cheah Biomedical Centre, Cambridge Biomedical Campus, Cambridge CB2 0AW, UK; 3 Department of Medical and Surgical Specialties, Radiological Sciences and Public Health, University of Brescia, 25121 Brescia, Italy; 4 Department of Protein Science, KTH Royal Institute of Technology, SciLifeLab, 171 65 Stockholm, Sweden; shaghayegh.bayati@scilifelab.se (S.B.); peter.nilsson@scilifelab.se (P.N.); 5 Department of Rheumatology and Clinical Immunology, University Medical Center Groningen, University of Groningen, 9713 GZ Groningen, The Netherlands; e.brouwer@umcg.nl; 6 Department of Pathology and Medical Biology, University Medical Center Groningen, University of Groningen, 9713 GZ Groningen, The Netherlands; p.heeringa@umcg.nl; 7 R&D Department, Hycult Biotech, 5405 PB Uden, The Netherlands; e.toonen@hycultbiotech.com (E.J.M.T.); m.beenes@certe.nl (M.B.); 8 Department of Pathology, Medical University of Vienna, 1090 Vienna, Austria; mjball@mjball.net (M.J.B.); andrew.rees@meduniwien.ac.at (A.J.R.); renate.kain@meduniwien.ac.at (R.K.)

**Keywords:** ANCA-associated vasculitis, biomarkers, autoantibody profiling, protein array, kinesins

## Abstract

ANCA-associated vasculitides (AAV) are rare autoimmune diseases causing inflammation and damage to small blood vessels. New autoantibody biomarkers are needed to improve the diagnosis and treatment of AAV patients. In this study, we aimed to profile the autoantibody repertoire of AAV patients using in-house developed antigen arrays to identify previously unreported antibodies linked to the disease per se, clinical subgroups, or clinical activity. A total of 1743 protein fragments representing 1561 unique proteins were screened in 229 serum samples collected from 137 AAV patients at presentation, remission, and relapse. Additionally, serum samples from healthy individuals and patients with other type of vasculitis and autoimmune-inflammatory conditions were included to evaluate the specificity of the autoantibodies identified in AAV. Autoreactivity against members of the kinesin protein family were identified in AAV patients, healthy volunteers, and disease controls. Anti-KIF4A antibodies were significantly more prevalent in AAV. We also observed possible associations between anti-kinesin antibodies and clinically relevant features within AAV patients. Further verification studies will be needed to confirm these findings.

## 1. Introduction

ANCA-associated vasculitides (AAV) are rare autoimmune diseases with a combined annual incidence of 25–33 cases per million people [1]. AAV is characterized by necrotizing inflammation of small blood vessels that affects potentially every organ, requiring prompt initiation of immunosuppressive treatment to limit organ damage. These diseases encompass a phenotypic spectrum, with two main clinico-pathological presentations: granulomatosis with polyangiitis (GPA), which involves granulomatous inflammation, often affecting the respiratory tract, and microscopic polyangiitis (MPA), which displays purely vasculitic features, with frequent kidney involvement [2,3,4].

Circulating autoantibodies directed against components of neutrophil and monocyte granules, named anti-neutrophil cytoplasmic antibodies (ANCAs), play a key role in the pathogenesis by inducing dysregulated activation of primed neutrophils and monocytes [3]. ANCAs recognize two main autoantigens, myeloperoxidase (MPO) and proteinase 3 (PR3). Autoantigen specificity is associated with clinical presentation: anti-MPO antibodies are identified in around 60% of patients with MPA and 20% of patients with GPA, while anti-PR3 antibodies are reported in around 75% of patients with GPA and 25% of patients with MPA [3]. ANCA testing plays a key role in the diagnosis of AAV [5], as well as in disease monitoring, and its titer may indicate disease activity during follow-up [6,7,8]. However, around 10% of patients with clinico-pathological features of AAV have no detectable ANCAs. Moreover, ANCA specificity does not fully account for the extreme heterogeneity in clinical presentation and organ involvement observed in AAV, and does not uniformly predict disease activity [3]. ANCAs can be found at low titers also in healthy individuals, further highlighting that their pathogenicity remains not completely understood [9]. Therefore, the identification of new autoantibodies that could improve the accuracy of diagnosis, monitoring, and subclassification of AAV is of great importance.

Recent studies have identified new autoantibodies in patients with AAV that could serve as potential biomarkers for the disease’s diagnosis and subclassification, such as anti-human lysosome-associated membrane protein-2 (hLAMP-2). Anti-hLAMP-2 antibodies have been detected in more than 70% of patients with AAV (including those with no detectable ANCAs), and their presence has been associated with more severe renal disease [10].

Other novel autoantibodies reported in AAV include anti-plasminogen antibodies, which have been hypothesized to increase thrombotic risk and renal damage [11], and anti-C5aR antibodies, low concentrations of which have been linked to disease activity and relapse [12].

In this study, we performed a broad autoantibody profiling of serum samples from patients with AAV as well as healthy and disease controls to search for novel autoantibodies associated with the presence of disease per se, disease subgroups, or disease activity. To achieve this goal, we applied our in-house developed protein array technology based on the collection of human protein fragments from the Human Protein Atlas project [13]. This well-established technology has already been successfully applied to profile the autoantibody repertoire in other autoimmune conditions [14,15] as well as in healthy individuals [16].

## 2. Results

### 2.1. Autoantibody Screening Revealed Reactivities to Kinesins in AAV Patients

**Figure 1:**
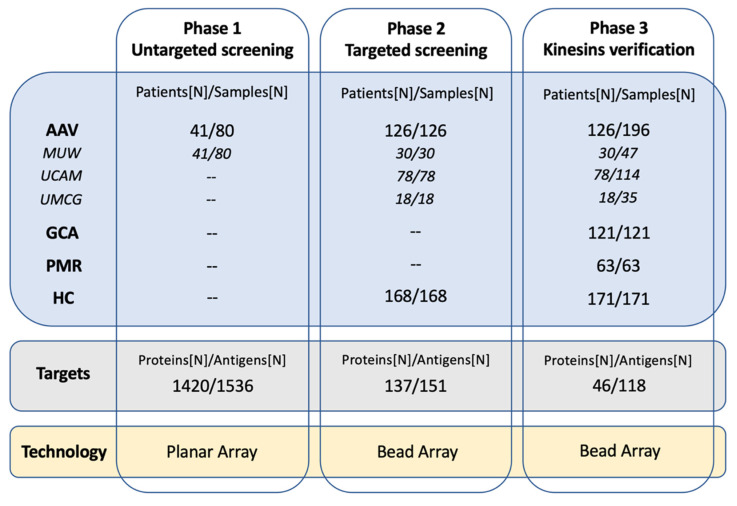
Study design. Phase 1: An untargeted screening on antigen planar array was performed to detect IgG against 1420 proteins in 80 samples from 41 patients. Thirty samples were collected at diagnosis, 33 at remission, and 17 at relapse. Phase 2: A bead array was generated with 151 protein fragments representing 137 proteins selected from phase 1 and the literature and used to test a multicentric sample set collected from 126 AAV patients at diagnosis (including the 30 samples at diagnosis tested in phase 1) and healthy controls. Phase 3: A verification phase was designed to screen IgG binding to 118 protein fragments representing 46 protein members of the kinesin superfamily and associated proteins. In this phase, the samples collected from AAV patients at diagnosis and included in phase 2 (N = 126) were tested together with matched samples collected at remission (N = 65) and relapse (N = 5) to evaluate longitudinal profiles. Moreover, healthy and disease (i.e., GCA and PMR) controls were included to evaluate the autoantibodies specificity. Acronyms: AAV, ANCA-associated vasculitis; GCA, giant cell arteritis; PMR, polymyalgia rheumatica; HC, healthy controls; MUW: Medical University of Vienna, Austria; UMCG: University Medical Center, Groningen, Netherlands; and UCAM: University of Cambridge, UK.

An initial untargeted screening on the planar array identified 40 proteins targeted by immunoglobulin G (IgG) in at least 2 out of 80 AAV samples. These proteins, together with 97 more selected from the literature, were included in a targeted antigen bead array used to screen IgG in 126 serum samples collected from AAV patients at diagnosis and 168 samples from healthy individuals (Figure 1 and Table 1). When available, we included multiple protein fragments for each protein to ensure the largest possible coverage of the protein sequence, thereby generating a bead array with 151 protein fragments representing 137 unique Uniprot IDs (Supplementary Table S2). Overall, we identified IgG binding to 117 out of the 151 antigens (77%) across all tested samples. The median number of autoantibodies detected per single sample (i.e., autoantibody load) was five (range 0–14). AAV patients and healthy controls had similar autoantibody loads (Supplementary Figure S1a). The autoantibody load was also not affected by sex, age, or by treatment of AAV, and it did not correlate with the BVAS score (Supplementary Figure S1b–e). AAV patients testing positive for anti-MPO ANCA showed a tendency to higher autoantibody load compared to anti-PR3 ANCA positive patients (Wilcoxon test, *p* = 0.078), while no difference was detected when comparing MPA and GPA patients (Supplementary Figure S1f,g).

When considering specific antibodies, anti-KIF4A (aa 376–461) showed higher prevalence in AAV (5%, 6 reactive samples out of 125—one AAV sample was excluded for technical reasons) compared to the healthy individuals (1%, 1 reactive sample out of 168) (Fisher’s exact test, *p* = 0.045). Anti-KIF15 (aa 671–748) also showed a tendency to higher prevalence in AAV (16%, 20 positive samples out of 125) compared to healthy controls (10%, 17 positive samples out of 168) (Fisher’s exact test, *p* = 0.156). The results for anti-KIF4A (aa 376–461) and anti-KIF15 (aa 671–748) were also replicated in a proof-of-concept analysis with ELISA assays generated using the same protein fragments used in the antigen bead array. The data showed that we could replicate the bead array results of four samples from AAV patients seropositive for either anti-KIF4A (aa 376–461) or anti-KIF15 (aa 671–748) and two samples seronegative for both antibodies. The test also included a selection of healthy controls that showed a trend with lower intensity than the seropositive samples (Supplementary Figure S2).

### 2.2. Anti-Kinesin Antibodies at Higher Prevalence in Anti-MPO Positive and MPA Patients

Based on the above-mentioned results, showing two anti-kinesin antibodies at higher prevalence in patients with AAV compared to healthy individuals, we designed a new array to screen autoantibodies targeting the whole kinesin family of motor proteins in longitudinal samples collected from AAV patients, as well as single timepoint samples from healthy and disease controls. The bead array included 118 protein fragments representing 41 out of 45 kinesin (*KIF*) genes, ensuring coverage for all the 15 kinesin subfamilies. Moreover, we included four kinesin light chains (KLC 1–4), which are part of kinesin-1 and are responsible for recognizing and binding cargo proteins [17], and 1 kinesin-associated protein (KIFAP3) which has no motor functions but is associated with KIF3A and B [18] (Supplementary Tables S3 and S4). We applied the new array to test 196 serum samples collected from 126 AAV patients at diagnosis (N = 126), remission (N = 65), and relapse (N = 5), 121 serum samples from patients with giant cell arteritis (GCA), 63 serum samples from patients with polymyalgia rheumatica (PMR), and 171 serum samples from healthy individuals. GCA and PMR samples were included as relevant disease controls with the aim of evaluating the disease specificity of the identified autoantibodies.

In this paragraph we report the results of the comparison between 126 AAV samples collected at diagnosis, 121 GCA, 73 PMR, and those from 171 healthy individuals, while results related to the longitudinal samples are reported in the following paragraph.

Initially, we performed a PCA analysis to test whether different diagnosis or sample provenience could affect the data distribution. The analysis excluded any major batch effects (Supplementary Figure S3).

The anti-kinesin autoantibody load was similar for AAV at diagnosis, GCA, PMR, and for the healthy control groups (Supplementary Figure S4a). Age distribution was different between AAV and the disease controls, but our data showed no correlation between age and autoantibody load (Supplementary Figure S4b,c). The median anti-kinesin load was four autoantibodies in females and five autoantibodies in males (Wilcoxon’s test, *p* = 0.038). As in phase 2, the autoantibody load was not significantly affected by immunosuppressive treatment, even though at this stage we could see a trend with higher levels in non-treated samples (Wilcoxon’s test, *p* = 0.068) (Supplementary Figure S4d,e). As in phase 2 of the study, we detected a trend with higher anti-kinesin autoantibody load in anti-MPO positive AAV patients compared to anti-PR3 patients (Wilcoxon’s test, *p* = 0.052), but no difference when comparing MPA and GPA patients (Supplementary Figure S4f,g).

**Figure 2:**
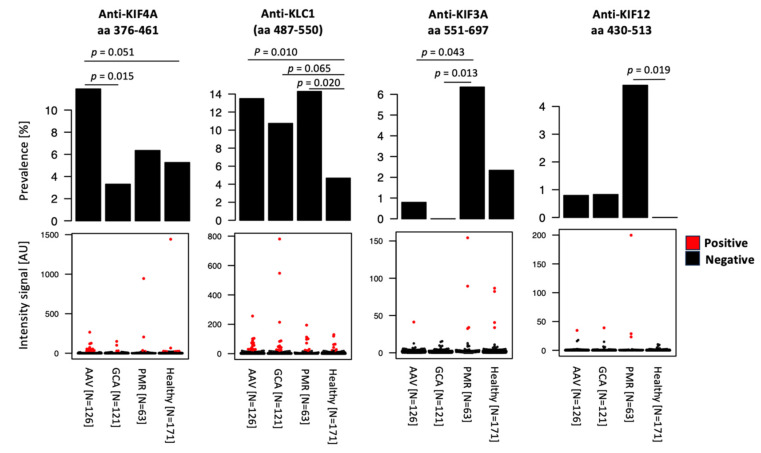
Anti-kinesin antibody seroprevalence in AAV at diagnosis compared to controls. Bar plots and dot plots represent, respectively, the prevalence and intensity signals detected for the selected autoantibodies. Each dot in the dot plots represents one individual; red dots indicate the individuals classified as seropositive for the autoantibody. *p*-values refer to Fisher’s exact test. Acronyms: AAV, ANCA-associated vasculitis; GCA, giant cell arteritis; and PMR, polymyalgia rheumatica.

IgG reactivity was detected in 85/118 fragments (72%) in AAV patients at diagnosis, 81/118 (69%) for GCA patients, 67/118 (57%) for PMR patients (disease controls), and 93/118 (79%) for the healthy controls. Prevalence above 10% was reported for 10 autoantibodies in the AAV and GCA patient groups, and for 14 in both PMR and healthy controls. Maximum prevalence ranged between 38% and 48% in the different sample groups (Supplementary Figure S5). Overall, the most prevalent antibodies were anti-KIF18a (aa 536–628) (59%), anti-KIF20B (aa 703–851) (57%), and anti-KIF3B (aa 400–450) (40%), with all three being included among the five most prevalent in AAV patients and control groups. On the other hand, anti-KIF4A (aa 376–461) showed significantly higher prevalence in AAV and anti-KLC1 (aa 487–550) in AAV, GCA, and PMR, while anti-KIF3A (aa 376–461) and anti-KIF12 (aa 430–513) were both more prevalent in patients with PMR compared to GCA and other groups (Supplementary Figure S5 and Figure 2).

**Figure 3:**
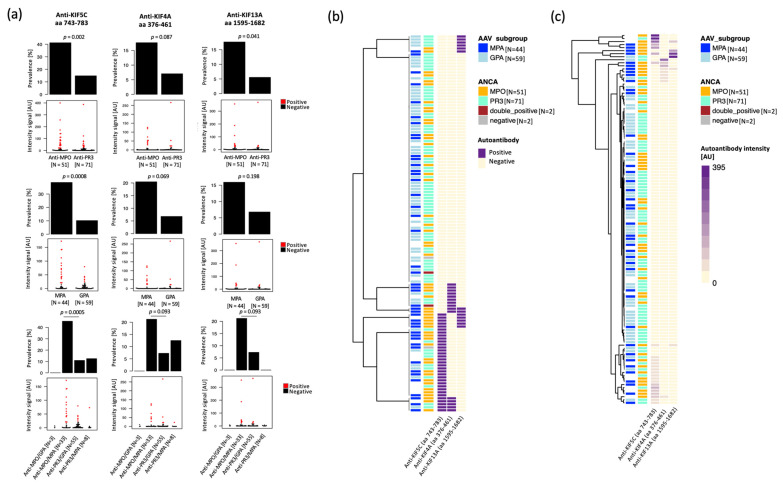
Anti-kinesin autoantibodies at higher seroprevalence in anti-MPO positive and MPA patients. (**a**) Bar plots and dot plots representing, respectively, the prevalence and intensity signals detected for autoantibodies selected during the comparison between AAV clinical subgroups. Each dot in the dot plots represents one individual, and the red color indicates the individuals passing the cutoff for seropositivity for a specific autoantibody. *p*-values refer to Fisher’s exact test. (**b**) The binary classification for the four antibodies of interest have been combined in a heatmap showing the distribution of the reactivities among the 126 AAV patients at diagnosis. (**c**) Heatmap showing the normalized intensities of the selected autoantibodies. Acronyms: AAV, ANCA-associated vasculitis; ANCA, anti-neutrophil cytoplasmic antibodies; anti-MPO, anti-myeloperoxidase antibodies; anti-PR3, anti-proteinase 3 antibodies; MPA. Microscopic polyangiitis; and GPA, granulomatosis with polyangiitis.

When comparing AAV clinical subgroups, antibodies binding to KIF5C (aa 743–783) showed an increased prevalence and median signal intensity in anti-MPO positive compared to anti-PR3 positive AAV samples (41% vs. 15%, *p* = 0.002). We could also detect a higher prevalence of anti-KIF5C (aa 743–783) antibodies in MPA compared to GPA patients (39% vs. 10%, *p* = 0.0008), with the largest difference detected when comparing anti-MPO positive MPA patients vs. anti-PR3 positive GPA patients (45% vs. 11%, *p* = 0.0005). Antibodies binding to KIF13A (aa 1595–1682) also showed a higher prevalence in anti-MPO positive AAV patients compared to anti-PR3 positive patients (18% vs. 6%, *p* = 0.041), and a trend with increased prevalence in MPA patients compared to GPA patients (16% vs. 7%, *p* = 0.198). In Figure 3, we report all the antibodies that showed at least 10 percentage units higher reactivity either in anti-MPO positive or anti-PR3 positive AAV patients, as well as for the anti-MPA vs anti-GPA comparison and the anti-MPO positive MPA patients vs anti-PR3 positive GPA patients’ comparison. Next to anti-KIF5C (aa 743–783) and anti-KIF13A (aa 1595–1682), anti-KIF4A (aa 376–461) also passed this cutoff and showed a trend with higher prevalence in anti-MPO positive and MPA patients (*p* = 0.093) (Figure 3a). None of the detected antibodies showed significantly higher or at least 10 percentage units higher prevalence in anti-PR3 positive and/or GPA patients.

Data shows that 64% (21/33) of the anti-MPO positive MPA patients were positive for at least one of the three antibodies, compared to 24% (13/55) of the anti-PR3 positive GPA patients (Fisher’s exact test, *p* = 0.0003). In the same way, 24% (8/33) of the anti-MPO positive MPA patients were positive for at least two out of the three selected autoantibodies compared to 2% (1/55) of the anti-PR3 positive GPA patients (Fisher’s exact test, *p* = 0.0014) (Figure 3b). A ROC curve analysis confirmed that, among the three antibodies, anti-KIF5C showed the best performance in terms of specificity and sensitivity in separating anti-MPO positive from anti-PR3 positive samples as well as MPA from GPA patients. The combination of the three autoantibodies separated a subgroup of anti-MPO positive MPA patients with 89.1% specificity and 57.6% sensitivity using intensity data, and 76.4% specificity and 63.3% sensitivity using binary data (Supplementary Figure S6).

To cover as much as possible of the protein sequence, we included several protein fragments for each of the proteins included in the kinesin autoantibody screening. However, for each of the three selected kinesins, only one protein fragment yielded informative results. KIF5C aa 743–783, KIF4A aa 376–461, and KIF13A aa 1595–1682 all represent parts of the kinesins’ stalk structure connecting the motor domain, which is responsible for the binding to the microtubules, to the globular tail, which is responsible for binding to the cargo.

With the aim of testing whether the presence of these antibodies can be due to molecular mimicry, we have performed a BLAST analysis to identify homologies between the sequences of the kinesin protein fragments targeted by the selected autoantibodies and viral (taxid:10239) and bacterial (taxid:2) proteins. The analysis did not identify any significant homology.

### 2.3. Anti-Kinesin Autoantibodies Show Association with Organ Involvement, While No Significant Variation Was Detected Based on Disease Activity

**Figure 4:**
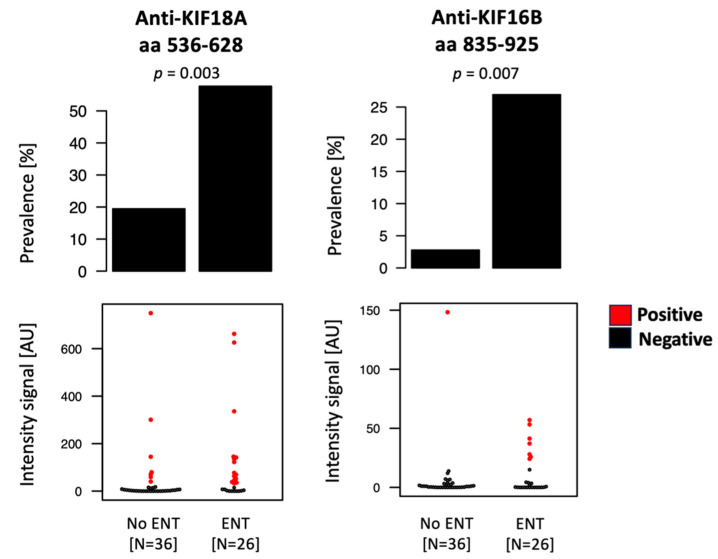
Anti-kinesin antibodies and organ involvement. Bar plots and dot plots representing, respectively, the prevalence and intensity signals for selected autoantibodies. Each dot in the dot plots represents one individual, and the red color indicates the individuals passing the cutoff for seropositivity for the specific autoantibody. Anti-KIF18A and anti-KIF16B showed increased prevalence in samples collected from AAV patients at diagnosis with ear–nose–throat (ENT) involvement. *p*-values refer to Fisher’s exact test.

Information on patterns of vasculitis organ involvement was available for 62 of the AAV patients from UCAM and MUW for which a sample was available at diagnosis (Supplementary Table S1). The presence of kidney, ear–nose–throat (ENT), or respiratory involvement did not show any association with increased autoantibody load (Supplementary Figure S7). On the other hand, we detected significantly higher prevalence of anti-KIF18A (aa 536–628) (Fisher’s exact test, *p* = 0.003) and anti-KIF16B (aa 835–925) (Fisher’s exact test, *p* = 0.007) antibodies in patients with ENT involvement (Figure 4). The symptoms recorded for AAV patients with ENT involvement include nasal discharge or crusting, epistaxis, sinus pain, hearing loss, recurrent otitis media, or stridor. No other autoantibody showed significantly increased prevalence associated with organ involvement.

We also investigated AAV longitudinal samples to evaluate whether anti-kinesin antibody prevalence varies depending on the disease activity status. At this scope, we focused on 65 of the 126 AAV patients (52%) for which matched samples were available at remission (65/65, 100%) and, for some, also at relapse (5/65, 8%). The analysis showed no difference, neither in terms of autoantibody load (Supplementary Figure S8) nor in the prevalence of single anti-kinesin antibodies at different stages of the disease.

## 3. Discussion

This study aimed at profiling the autoantibody repertoire in AAV using protein arrays. Through a 3-phase study design, we screened IgG reactivity in 1743 protein fragments, representing 1561 unique proteins in 229 samples collected from 137 patients with AAV, in addition to samples from 171 healthy individuals and 184 relevant disease controls, namely GCA and PMR. The main finding of our study is that antibodies targeting members of the kinesin protein family can be frequently detected in patients with AAV, as well as in healthy and disease controls. Kinesins are a superfamily of ATP-dependent motor proteins that, in mammals, are encoded by 45 genes (*KIF*s) and are phylogenetically subgrouped in 15 subfamilies, namely kinesin-1 to kinesin-14B. Kinesins are involved in a variety of functions as they take part, together with dyneins, in the trafficking of organelles, vesicles, and other types of cellular cargos [19]. Kinesins play essential roles in cell division [20,21] by being part of the mitotic spindle apparatus (MSA), which ensures the correct segregation of chromosomes [22], as well as in cell degranulation [23], in neuronal signaling, by transporting molecules from the cell body to synapsis [24], in ciliogenesis, and in cilia function. Their role in monogenic disorders is now emerging, and “kinesinopathies” are increasingly recognized as the cause of anomalies in the development of the brain, the kidneys, and the urinary tract, as well as syndromic phenotypes reminiscent of ciliopathies [25]. Genetic variation in kinesins has also been linked to susceptibility to autoimmunity: specifically, variants in *KIF21B* and *KIF5A* have been associated with multiple sclerosis in humans [26,27], while variants in *KIF1C* predispose mice to autoimmune orchitis [28]. This is in line with the recognized role of kinesins in the activation of immune cells and inflammatory responses [29].

Autoantibodies binding to kinesins have already been reported in autoimmune diseases. One of the main molecular targets of anti-MSA antibodies, a rare type of anti-nuclear antibodies (ANA), is KIF11, also known as HsEg5. Anti-MSA antibodies have been reported in connective tissue diseases, systemic lupus erythematosus (SLE), and rheumatoid arthritis (RA), but also in vasculitis [30,31,32]. While the MSA pattern is usually rare, sometimes it represents the only serological marker for patients that are negative for other types of ANA [33].

The clinical relevance of our findings remains to be completely elucidated. While we observed widespread anti-kinesin reactivity across all study subgroups, including healthy volunteers, we found some preferential associations between autoantibody specificities and diseases, such as reactivity against KIF4A and KLC1 in AAV. We also identified a few anti-kinesin autoantibodies specifically associated with anti-MPO-ANCA positivity and with vasculitis activity. We could speculate that specific patterns of anti-kinesins autoreactivity may predispose to AAV (or other autoimmune diseases) or modulate the disease phenotype in the setting of established disease. However, we must acknowledge that multiple testing, in the setting of a relatively limited sample size, may have led to false positive associations in our study. External validation will, therefore, be crucial to verify these potentially interesting observations and shed further light on clinical associations.

Another key finding was the autoantibody load (i.e., the total number of autoantibodies in a sample) similarity across all the main subgroups included in the study, and in particular between patients with AAV or other immune-mediated conditions and healthy controls. Although this may sound somewhat surprising at first, such observation is in line with other studies reporting widespread auto-reactivity in healthy individuals [34], including natural anti-MPO and anti-PR3 autoantibodies [9]. Nonetheless, further analyses to understand the significance and nature of anti-kinesin autoantibodies in healthy controls are advocated. On the other hand, the role played by autoantibodies in GCA and PMR is still not clear, and no link has been identified between autoantibodies and specific clinical features [35,36]. The identification of anti-kinesin autoantibodies in GCA and PMR in our study, therefore, warrants further investigation to establish their relation to clinical features.

Intriguingly, we also observed a tendency to higher autoantibody load in anti-MPO compared to anti-PR3 patients, which was not accounted for by the older age of MPO-ANCA positive patients. This observation, if confirmed in the future, may add to a growing body of evidence suggesting that anti-MPO and anti-PR3 AAV may be distinct diseases, with important differences between the two encompassing demographics, geographic distribution [37,38], genetic associations [39], organ involvement [40,41], and prognosis [40]. Also, anti-MPO AAV is reported to be associated with respiratory infections, but not anti-PR3 AAV [42]. The difference in autoantibody load between MPO- and PR3-AAV that we found may further reflect underlying pathophysiological differences and deserves further studies.

The multi-step study design made it possible to perform a broad unbiased screening of autoantibodies based on protein arrays, while adapting second-level analysis to the initial results. The inclusion of healthy controls, as well as disease controls, is another major point of strength, making it possible to better put the results into context.

The ELISA proof-of-concept experiments show the possibility of transferring the antigen-array assay into ELISA formats using the Human Protein Atlas protein fragments as capture molecules. This represents a strength as, at later stage in the validation of these autoantibodies as candidate biomarkers, an ELISA assay could be more easily implemented in clinical laboratories without requiring the acquisition of expensive equipment.

Important limitations need to be acknowledged too. First, as already mentioned, false positive results in the setting of multiple testing cannot be excluded, which makes our analysis on clinical associations highly exploratory. The intrinsic limitations linked to the technology platform that we adopted, namely arrays with recombinant protein fragments synthetized in *E. coli*, need to be recognized as well. It is well established that such an approach can lead to false negative results, especially in the case of epitopes with complex conformations or glycosylation patterns or not covered by the protein fragment sequence. On the other hand, our approach may also lead to the detection of conformational epitopes that, due to the protein fragment’s specific nature and folding, may not be present in the native conformation of the protein. Further verification of the identified autoantibodies using epitope mapping analysis and arrays with full-length proteins may be of help to highlight whether the identified epitopes are linear or reflect native conformational epitopes. Moreover, a finer epitope mapping analysis is needed to explore potential molecular mimicry that could link the identified antibodies to previous infections.

Another weakness of this study is the limited inclusion of longitudinal samples with heterogeneous time points across different individuals. A more systematic longitudinal sampling will be needed in future works to better assess the correlation between disease activity and autoantibody levels.

To conclude, through a broad autoantibody discovery screening, we identified a high prevalence of antibodies reactive to kinesins in samples from AAV patients, as well as healthy volunteers and disease controls with other immune-mediated diseases. We observed some intriguing associations between patterns of anti-kinesin reactivity and these other immune-mediated diseases, as well as clinically relevant features within AAV patients. Further studies on independent cohorts will be needed to validate these findings and further delve into the clinical relevance of anti-kinesin autoantibodies, both in the context of health and of disease.

## 4. Materials and Methods

### 4.1. Study Group

The present study includes 137 patients with ANCA-associated vasculitis (AAV), of which 41 patients were recruited at the Medical University of Vienna (MUW), 78 at the University of Cambridge (UCAM), and 18 at the University Medical Center of Groningen (UMCG). A total of 229 serum samples were collected, including samples collected at diagnosis (N = 126), during remission (N = 83), and at relapse (N = 20). Available clinical information includes age, sex, clinical phenotype (MPA or GPA), ANCAs serology data (anti-MPO and anti-PR3 antibodies), organ involvement, disease activity—captured using the BVAS score [43]—and treatment (Table 1 and Supplementary Table S1). As healthy controls, we included 171 serum samples from volunteers with no known systemic diseases. Moreover, 121 serum samples from patients with giant cell arteritis (GCA), and 63 serum samples from patients with polymyalgia rheumatica (PMR) were included in the study as disease controls to test the specificity of autoantibodies detected in AAV (Table 1).

Informed consent covering the presented research was collected at the site of sample collection for all individuals included in this study. The study was approved by the Cambridge Local Research Ethics Committee (ref. nr. 04/023, 08/H0306/21, 08/H308/176), by the Ethics Committee at the Medical University of Vienna Ethik Kommission Medizinischen Universität Wien (ref. nr. 1089/2012 and 2273/2016), and by the Institutional Review Board of the University Medical Center Groningen (UMCG) (ref. nr. METc2012/375 for healthy controls, METc2010/222 for GCA patients, and METc2012/151 for AAV patients).

### 4.2. Study Design

The study was carried out in three phases (Figure 1). Initially, an untargeted screening was performed using antigen planar arrays on 80 serum samples collected from 41 patients at diagnosis, remission, and relapse and provided by MUW (Phase 1). The aim of this screening was to select proteins targeted by IgG antibodies in AAV. The selection was complemented with proteins identified in the literature as related to AAV and used to generate a first targeted antigen bead array used in the second phase of the study to screen 126 serum samples collected at diagnosis from the same number of AAV patients provided by MUW (including 30 samples tested in phase 1), UCAM, and UMCG. At this stage, we also included samples from 168 healthy individuals (Phase 2). Based on results from the second phase, a verification screening was designed to detect autoantibodies targeting proteins of the kinesin superfamily. A new bead array was generated and used to test the samples from AAV patients at diagnosis included in phase 2, but also matched samples collected at remission and at relapse, as well as healthy and disease controls (i.e., GCA and PMR) (Phase 3). The data analysis focused on identifying single autoantibodies or combinations that could be associated with AAV per se, AAV subgroups (i.e., anti-MPO and anti-PR3 along with MPA and GPA), organ involvement, or disease activity.

### 4.3. Antigens

The antigen arrays used for this screening study have been generated using protein fragments (40–100 amino acids) produced in *E.coli* and available at the Human Protein Atlas [44]. The fragments were designed in a gene-centric manner to represent the sequence region with the lowest homology to all other human proteins [45].

### 4.4. Untargeted Autoantibody Screening Using Planar Antigen Array

Eighty AAV samples from 41 patients at different stages of the disease (namely, diagnosis/presentation, remission, and relapse), on and off treatment, were tested using in-house developed antigen planar arrays including 1516 antigens, representing 1420 unique proteins (Figure 1). The assay was performed as previously described [46]. Briefly, each sample was diluted 1:100 in assay buffer containing PBS 0.1% (*v*/*v*) Tween20 (Thermo Fisher Scientific, Waltham, MA, USA), 3% bovine serum albumin (Saveen Werner, Limhamn, Sweden), 5% (*v*/*v*) skim milk powder (Sigma-Aldrich, St. Louis, MO, USA), and 160 µg/mL His_6_ABP. After pre-incubation of the diluted sample for 15 min at room temperature, 100 μL were applied on each microarray slide and incubated for 1 h at room temperature. Sample excess was eliminated with PBS-T 0.1%. The array was then incubated for 1 h with 1:40,000 hen anti-His6ABP IgY (Immunotech HPA, Stockholm, Sweden), which allows the detection of the microarray spots. After additional washes with PBS-T 0.1%, the detection was performed with fluorescently labeled goat anti-chicken IgY Alexa Fluor^®^ 555 (A21437, Invitrogen, Waltham, MA, USA) and goat anti-human IgG (H + L) Alexa Fluor^®^ 647 (A21445, Life Technologies, Carlsbad, CA, USA), diluted 1:15000. Readout was performed with a laser scanner (InnoScan^®^ 1100, Innopsys, Chicago, IL, USA), followed by image analysis performed by GenePix Pro 5.1 (Molecular Devices LLC, San Jose, CA, USA).

### 4.5. Targeted Autoantibody Screening and Anti-Kinesin Autoantibody Verification by Antigen Bead Array

We designed two tailored bead-based antigen arrays. The first one included 151 antigens selected from the planar array analysis and literature search (Supplementary Table S2) and was applied in study phase 2, while the second one was generated using 118 protein fragments representing 46 unique kinesin (*KIF*s) genes, kinesin-light chain (*KLC*s) genes and kinesin-associated protein (*KIFAP3*) gene (Supplementary Tables S3 and S4) and was applied in study phase 3 (Figure 1).

Both bead arrays were generated by immobilizing the different antigen types on the surface of uniquely color-coded magnetic beads (bead identities) (MagPlex, Luminex Corp., Austin, TX, USA), followed by mixing all bead identities to generate the suspension bead array. Plasma and serum samples were tested as previously described [15]. In brief, each sample was diluted 1:250 in assay buffer containing PBS-T 0.05%, 3% (*w*/*v*) BSA (Saveen Werner, Limhamn, Sweden), 5% (*w*/*v*) skim milk powder (Sigma-Aldrich, St. Louis, MO, USA), and supplemented with 160 µg/mL His6ABP, then pre-incubated for 1 h at room temperature to quench any cross-reactivity to the His6ABP tag included in the antigen sequence. The samples were then incubated with the bead array for 2 h at room temperature. After washing away all sample in excess by using PBS-T 0.05%, the autoantibody-antigen complexes were cross-linked with 0.2% paraformaldehyde. The IgG bound to the antigens were detected with 0.4 µg/mL R-PE conjugated anti-human IgG detection antibody (H10104, Invitrogen, Waltham, MA, USA) for 30 min at room temperature. The readout was performed with FLEXMAP 3D^®^ instrument. (Luminex Corp., Austin, TX, USA).

### 4.6. ELISA Assay

Patient and healthy control samples were assessed using two newly developed ELISAs for the detection of either anti-KIF4A (aa 376–461) or anti-KIF15 (aa 671–748) IgG autoantibodies in serum or plasma. Microtiter wells (Nunc maxisorp cat# 468667, Thermo Fisher Scientific, Waltham, MA, USA) were coated with recombinant KIF4A (aa 376–461) or KIF15 (aa 671–748) (4 µg/mL) (produced in *E.coli* and available at the Human Protein Atlas) at 4 °C overnight. After blocking (1x PBS, 1%BSA) for 1–1.5 h, wells were washed twice (1x PBS, 0.05% Tween20), preserved (2*20 sec. in 200 µL 0.1%BSA/5%SkimMilk/PBS), sealed, and stored at 4 °C until further use.

For both anti-KIF4A and anti-KIF15, two seropositive and two seronegative samples, identified using the antigen array, were selected and tested next to ten healthy controls. All samples were selected from the UCAM cohort. In detail, each sample was diluted 1:50 in assay buffer containing PBS-T 0.05%, 3% (*w*/*v*) BSA, and 5% (*w*/*v*) skim milk powder and supplemented with 200 µg/mL His6ABP. Diluted samples were pre-incubated in buffer for 1 h at room temperature to block any tag-binding proteins. Subsequently, pre-incubated samples were added to the pre-coated wells and incubated for 1h at room temperature while shaking at 250 rpm. After washing (4 times in 1x PBS, 0.05% Tween20), wells were incubated with 0.03 µg/mL biotinylated goat anti-human IgG (FC specific) (Sigma, cat. nr. I2136) in PBS 0.1% BSA for 1 h at room temperature. After washing, wells were incubated with 33 pg/mL Streptavidin-HRP (Fisher Scientific) for 1 h at room temperature. Wells were washed again and tetramethylbenzidine (TMB) substrate was added. This started an enzymatic reaction, thereby producing a colored product which could be measured. The reaction was stopped after 30 min by adding oxalic acid and the absorbance at 450 nm was measured using a spectrophotometer. All obtained signals were specific for either anti-KIF4A or anti-KIF15 IgG autoantibodies as no signals were obtained in the absence of the recombinant proteins (non-coated wells). Anti-KIF4A or anti-KIF15 autoantibody positivity was defined by an optical density value measured at 450nm (OD450) in samples exceeding the cutoff value, represented by the mean +3SD in healthy controls.

### 4.7. Data Analysis

Data analysis and visualization was performed with R studio version 4.0.4.

The planar and bead array intensity data were transformed into binary values (reactive/non-reactive) by setting a cutoff based on the formula *median(x_n_) + nMAD*, where *median(x_n_)* is the median intensity signal across all antigens in each sample, *MAD* represents the median absolute deviations (MAD) from the sample median, and the factor *n* is determined differently for planar and bead array. For planar array the *n* factor was determined based on the array specific background, while for bead array it was determined based on the distribution of the intensity signals for each antigen across all samples [15]. Therefore, assay specific cutoffs were set to *median + 30x MAD* for the untargeted screening (study phase 1), *median + 50x MAD* for the first targeted screening (study phase 2), and *median + 20x MAD* for the kinesin screening (study phase 3). An antibody was defined as *positive* in a specific sample when passing the sample-specific cutoff of reactivity.

In addition to transformation into binary values, the bead array intensity signals were also normalized for sample and antigen specific backgrounds using the formula *nMAD = (x_i_-median(x_n_))/MAD(x_n_)*, where *nMAD* represents the number of median absolute deviations (MAD) from the sample median of each single intensity signal, *x_i_* is the raw intensity signal of each single protein fragment in each sample, *median(x_n_)* is the median intensity signal across all antigens in each sample, and *MAD(x_n_)* is the MAD of the intensity signal across all antigens in each sample. The normalized intensities were used to generate the dot plots included in this paper.

Fisher’s exact test was applied to compare the prevalence of seropositivity to specific autoantibodies in AAV patients and the control groups, as well as in AAV patient subgroups. Mann–Whitney–Wilcoxon test and Kruskal–Wallis test were applied to compare the total number of autoantibodies (i.e., autoantibody load) across two or more sample groups.

Heatmaps and unsupervised hierarchical cluster analysis were applied to identify autoantibody signatures associated with AAV clinicopathological (MPA/GPA) and serological (anti-MPO/anti-PR3) subgroups. Principal component analysis (PCA) was performed to evaluate data variation based on sample type and provenience. Receiving operating characteristic (ROC) curve was applied to evaluate the performance of single autoantibodies and combinations in separating patient subgroups. The area under the curve (AUC) was calculated and bootstrap method applied to calculate difference between curves.

## Figures and Tables

**Table 1:** Sample cohort and demographics.

Demographics	AAV	GCA	PMR	Healthy
n (individuals/samples)	137/229	121/121	63/63	171/171
Provider ^a^				
MUW	41/80	-	-	-
UMCG	18/35	121/121	63/63	78/78
UCAM	78/114	-	-	93/93
Age, median (range)	62 (19–85) ^b^	69 (49–89)	69 (50–84)	64 (21–86)
Sex, *n* (%)				
Male	73 (53)	42 (35)	23 (37)	72 (42)
Female	64 (47)	79 (65)	40 (63)	99 (58)

^a^ MUW: Medical University of Vienna, Austria; UMCG: University Medical Center, Groningen, the Netherlands; and UCAM: University of Cambridge, UK. ^b^ Age at first encounter available for 134 out of 137 AAV patients.

## Data Availability

Data and code cannot be shared publicly as they contain sensitive personal information which is protected by the GDPR. Data and code are available on request from the SciLifeLab Data Repository (https://www.scilifelab.se/data/repository/) for researchers who meet the criteria for access to sensitive personal data.

## Supplementary Materials

The following supporting information can be downloaded at: https://www.mdpi.com/article/10.3390/ijms242015341/s1.
