# Supplementary material for: Autoantibody Profiling and Anti-Kinesin Reactivity in ANCA-Associated Vasculitis
Source: Int J Mol Sci. 2023 Oct 19;24(20):15341. doi: 10.3390/ijms242015341 (PMC10607136; doi:10.3390/ijms242015341)
Supplement: Supplementary file 1 [file ijms-24-15341-s001.zip › ijms-2637071-supplementary.pdf]

Supplementary Material

Supplementary Figures and Legends

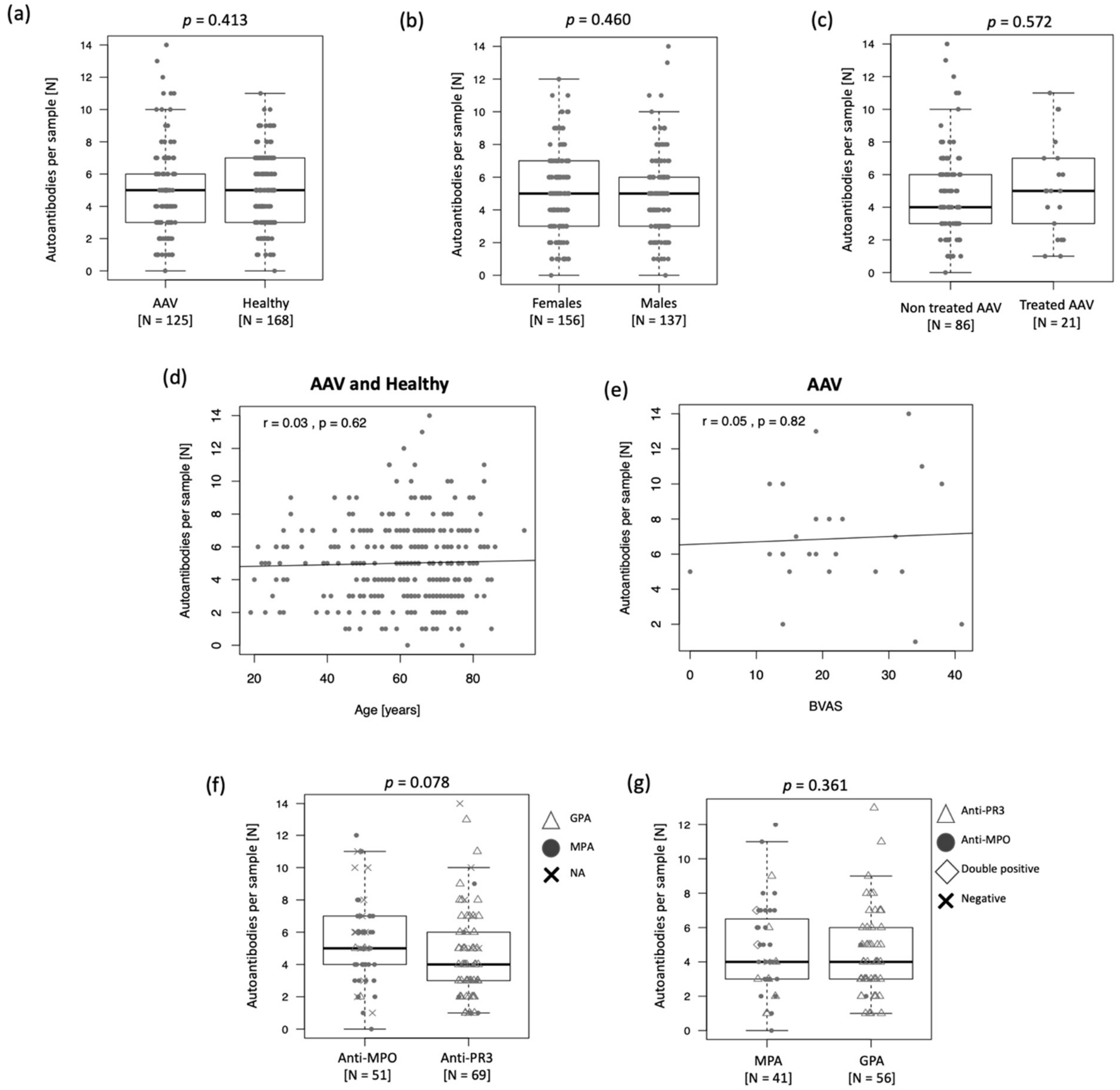

Supplementary Figure S1. Autoantibody load in study phase 2. (a) AAV versus healthy individuals. One individual was excluded from the analysis for technical reasons, so the total AAV samples is 125 instead of 126. (b) Males versus females across the whole cohort (AAV plus healthy controls). (c) AAV treated versus untreated. (d) Correlation between age and autoantibody load across the whole cohort. (e) Correlation between autoantibody load and BVAS score in AAV patients. (f) anti-MPO positive versus anti-PR3 positive AAV patients. (g) microscopic polyangiitis (MPA) versus granulomatosis with polyangiitis (GPA). In panels a-c and f-h Mann-Whitney-Wilcoxon test was applied to compare the distributions. Panels d-e report results for Pearson´s correlation.

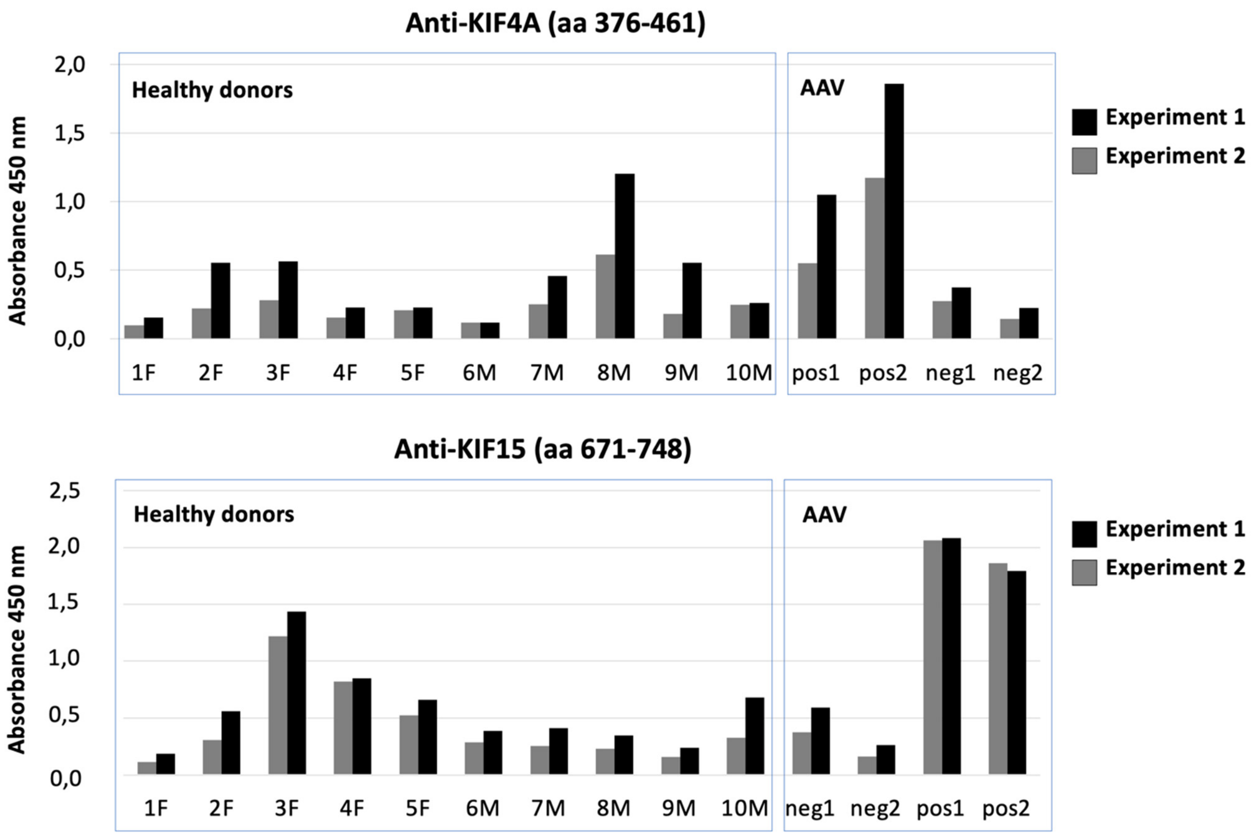

Supplementary Figure S2. ELISA test results for anti-kinesins identified in study phase 2. Two ELISA tests were generated using the same protein fragments used on bead-arrays and representing KIF4A (aa 376-461) and KIF15 (aa671-748). Samples included in the analysis were selected from the UCAM cohort. Each assay includes 14 serum samples collected from 10 healthy controls (numbered 1-10; M = male, F = female) plus 4 serum samples from AAV patients defined as seropositive (pos1 and pos2) or seronegative (neg1 and neg2) for the tested autoantibody based on the antigen array analysis. While the 10 healthy controls and the 2 seronegative AAV samples are the same for both ELISAs (anti-KIF4A and anti-KIF15), the seropositive AAV samples were selected specifically for each test. Bars represent the absorbance of two replicate experiments performed in different days.

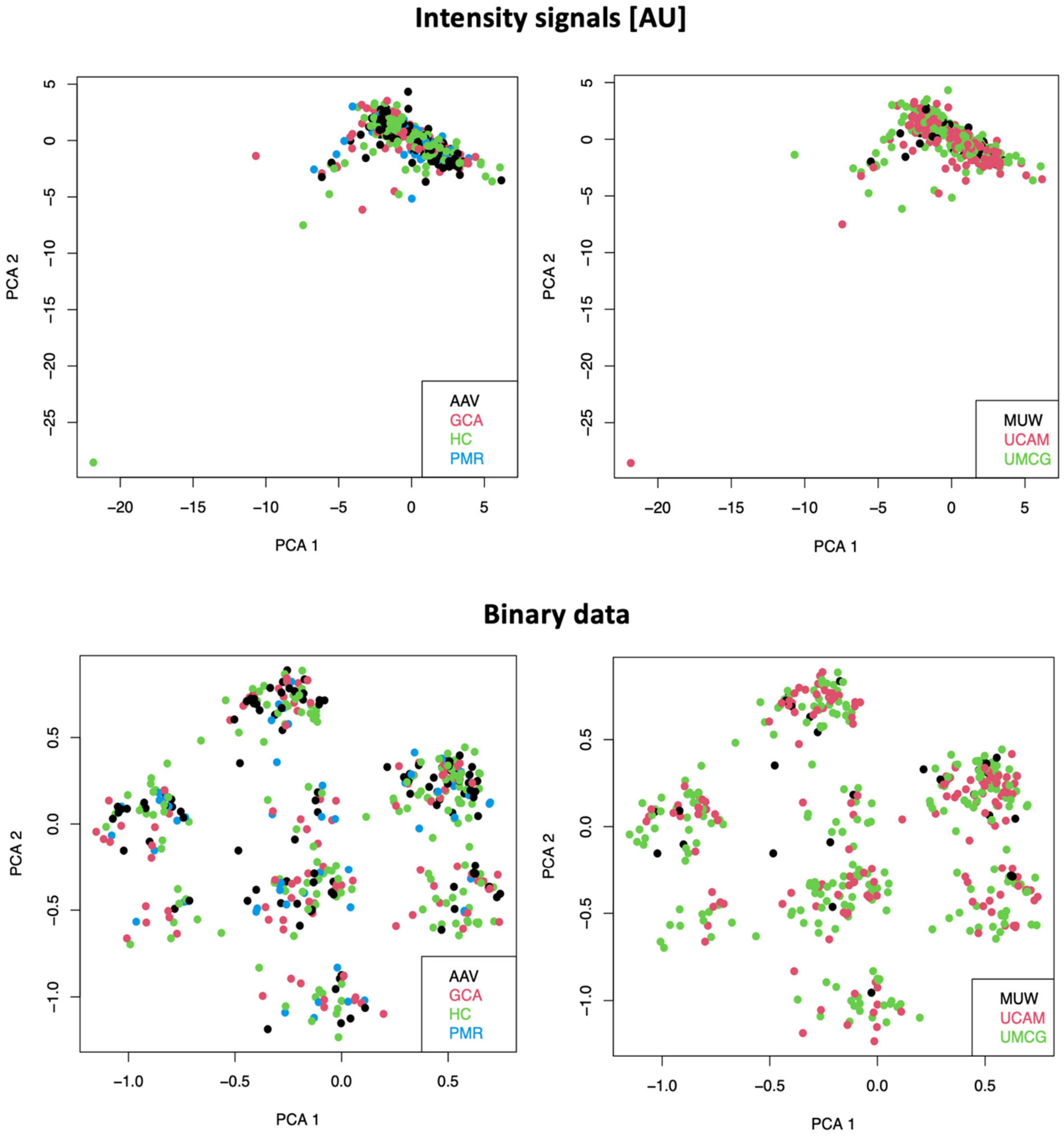

Supplementary Figure S3. Principal component analysis (study phase 3). PCA analysis was run based on normalized intensity signals (plots at the top) and binary data (plots at the bottom) for AAV samples at diagnosis (N=126), GCA (N=121), PMR (N=63), and healthy controls (N=171). Each dot in the plots represent one sample. Samples have been colored based on diagnosis (plots on the left) and clinical center of origin (plots on the right). Acronyms: AAV, ANCA-associated vasculitis; GCA, giant cell arteritis; PMR, polymyalgia rheumatica; HC, healthy controls; MUW, Medical University of Vienna; UMCG, University Medical Center Groningen; UCAM, University of Cambridge.

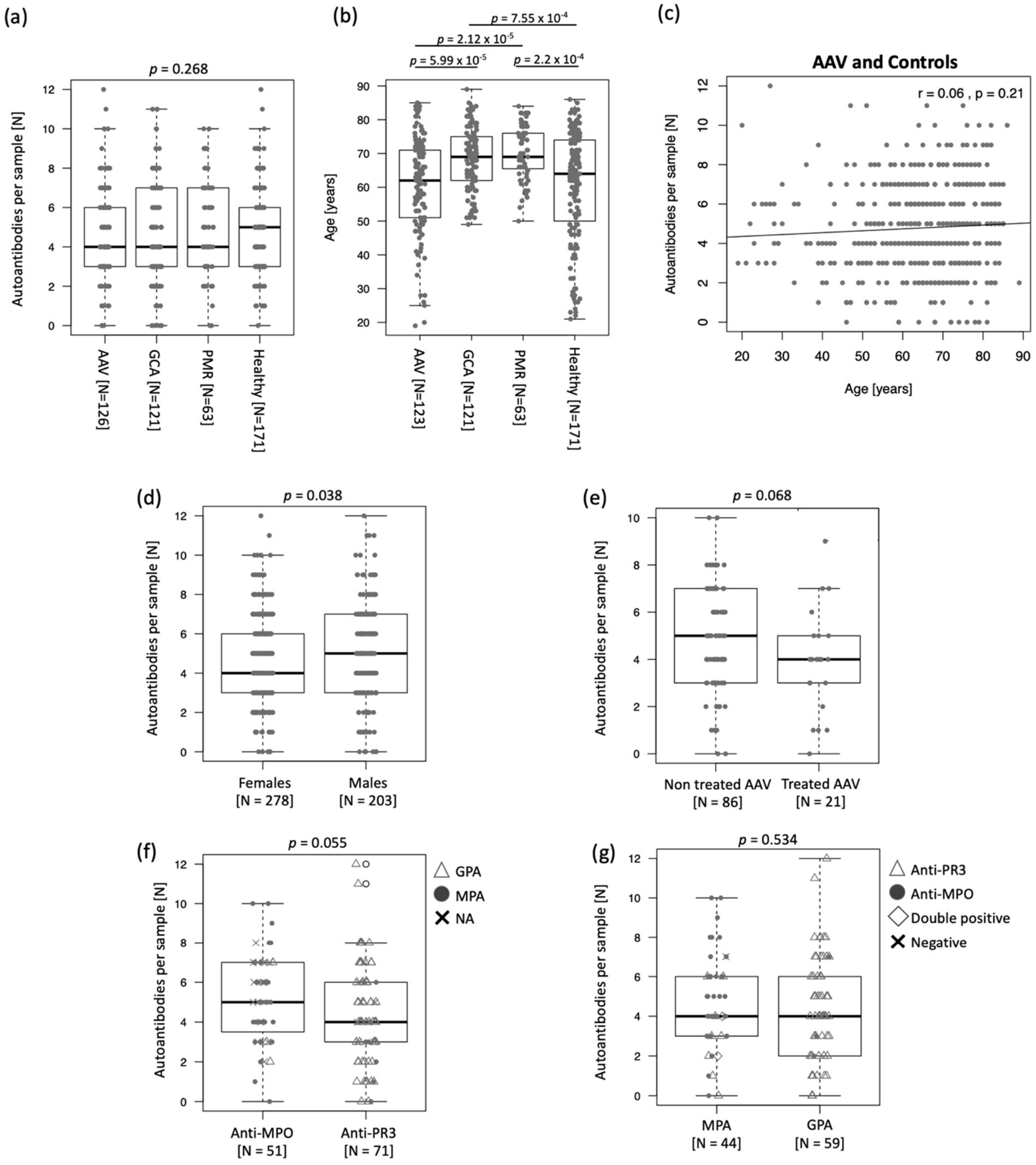

Supplementary Figure S4. Anti-kinesin autoantibody load. (a) Autoantibody load of AAV at diagnosis, GCA, PMR and healthy individuals. (b) Age of AAV at diagnosis, GCA, PMR and healthy individuals. (c) Pearson´s correlation between autoantibody load and age across the test group. (d) Autoantibody load by sex across the whole sample cohort. (e) Autoantibody load in treated and untreated AAV at diagnosis. (f) Autoantibody load in anti-MPO positive versus anti-PR3 positive AAV, and (g) in MPA and GPA patients. The p-values in the panels refer to: (a) Kruskal-Wallis test; (b) Kruskal-Wallis post-hoc Dunn-test; (d-g) Mann-Whitney-Wilcoxon test.

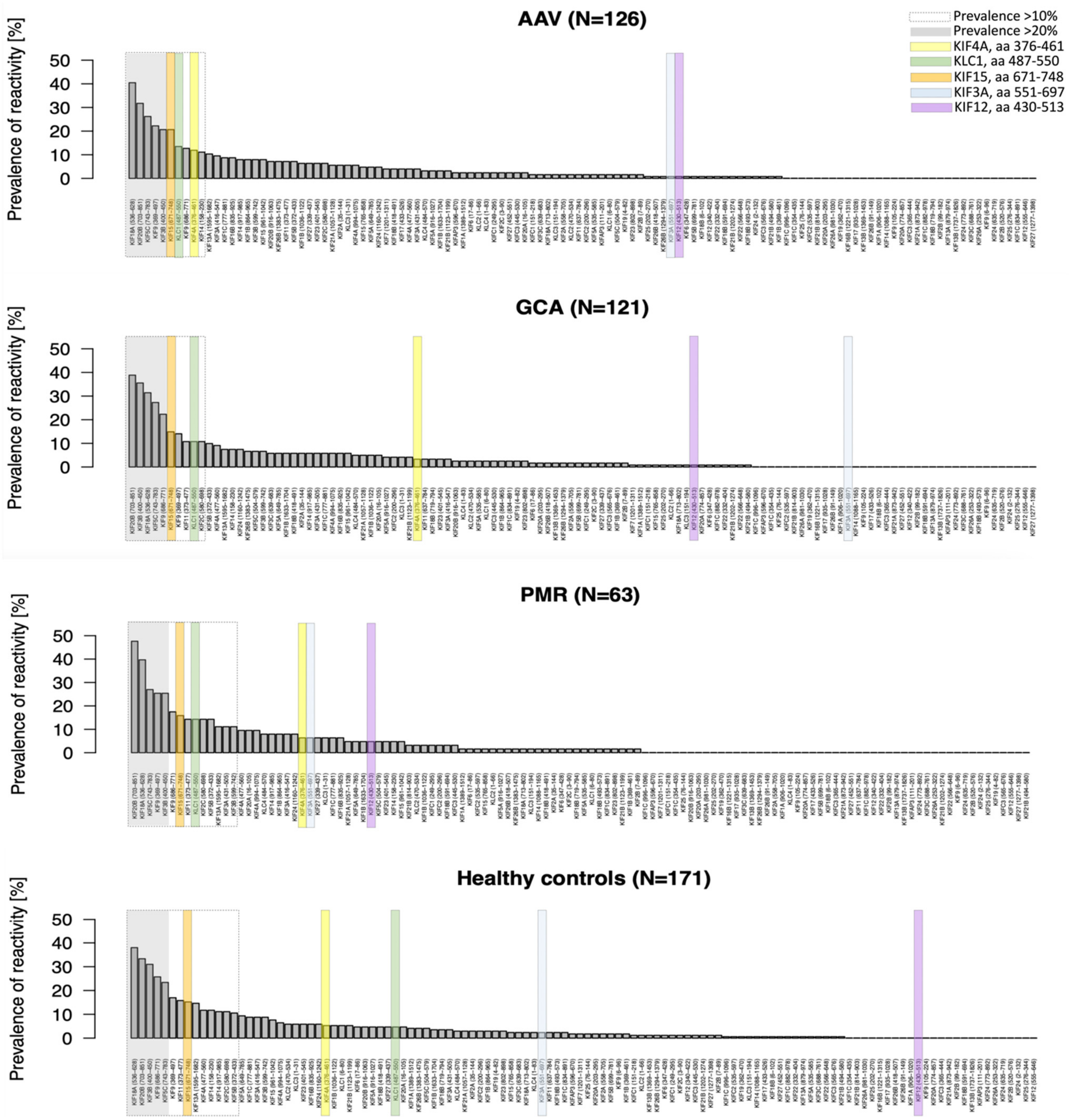

Supplementary Figure S5. Anti-kinesin autoantibody prevalence in AAV at diagnosis, GCA, PMR, and healthy controls. The barplots show the prevalence of reactivity towards each of the 118 kinesin representing antigens in patients and control groups. Acronyms: AAV, ANCA-associated vasculitis; GCA, giant cell arteritis; PMR, polymyalgia rheumatica.

(a)

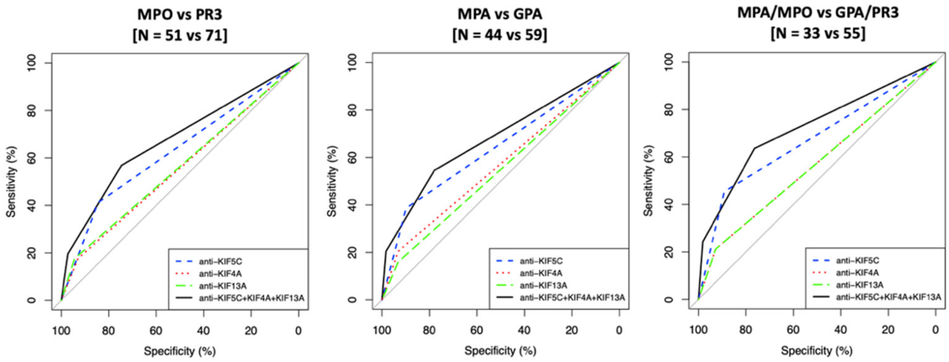

Antibody panel	MPO vs PR3				MPA vs GPA				MPA-MPO vs GPA-PR3			
	AUC	3 vs 1 antigen [p-value]	Sensitivity [%]	Specificity [%]	AUC	3 vs 1 antigen [p-value]	Sensitivity [%]	Specificity [%]	AUC	3 vs 1 antigen [p-value]	Sensitivity [%]	Specificity [%]
Anti-KIF5C	62.8	0.129	41.2	84.5	64.2	0.258	38.6	89.8	67.3	0.192	45.5	89.1
Anti-KIF4A	55.3	0.003	17.6	93.0	56.8	0.007	20.5	93.2	57.0	0.002	21.2	92.7
Anti-KIF13A	56.0	0.004	17.6	94.4	54.6	0.001	15.9	93.2	57.0	0.001	21.2	92.7
Anti-KIF4A+KIF5C+KIF13A	67.4	-	56.9	74.6	68.0	-	54.5	78.0	72.3	-	63.6	76.4

(b)

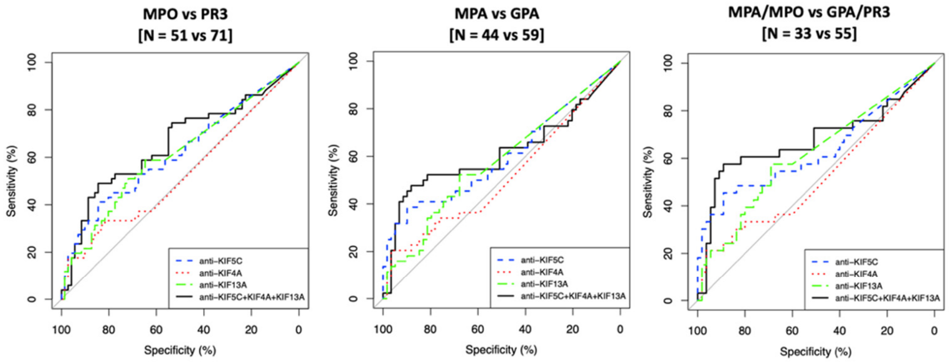

Antibody panel	MPO vs PR3				MPA vs GPA				MPA-MPO vs GPA-PR3			
	AUC	3 vs 1 antigen [p-value]	Sensitivity [%]	Specificity [%]	AUC	3 vs 1 antigen [p-value]	Sensitivity [%]	Specificity [%]	AUC	3 vs 1 antigen [p-value]	Sensitivity [%]	Specificity [%]
Anti-KIF5C	62.0	0.346	41.2	84.5	60.1	0.760	38.6	89.8	63.3	0.406	45.5	89.1
Anti-KIF4A	52.9	0.014	33.3	81.7	51.5	0.071	22.3	96.6	52.0	0.018	21.2	96.4
Anti-KIF13A	61.3	0.389	58.8	64.8	57.9	0.549	52.3	67.8	61.6	0.357	57.6	69.1
Anti-KIF4A+KIF5C+KIF13A	65.7	-	49.0	84.5	61.5	-	47.7	88.1	67.7	-	57.6	89.1

Supplementary Figure S6. ROC curve analysis of single and combined anti-kinesin autoantibodies. Curves and performance (in tables) of each selected autoantibody and their combination in separating AAV patients classified in anti-MPO positive and anti-PR3 positive, MPA and GPA, and the combination of the subgroups. (a) Refers to the analysis performed with binary data (seropositive/seronegative), while (b) is based on normalized intensities for anti-KIF5C (aa 743-783) and anti-KIF4A (aa 376-461). AUC, area under the curve; p-value, comparison between AUC of curve obtained by using only one autoantibody and all three of them in combination.

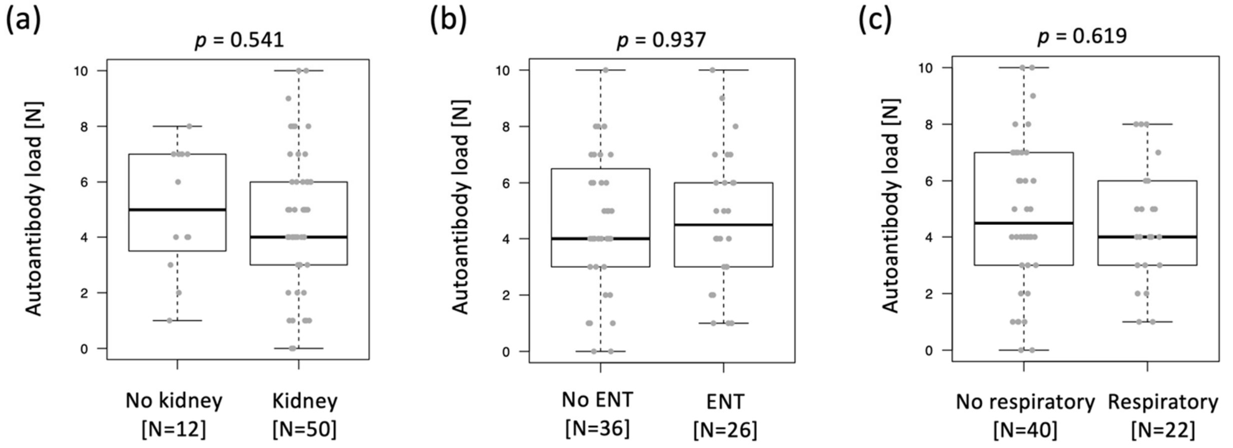

Supplementary Figure S7. Anti-kinesin autoantibody load in AAV and organ involvement. (a) Autoantibody load in serum samples collected at diagnosis for AAV patients with available data regarding (a) kidney, (b) ear-nose-throat (ENT) and (c) respiratory involvement. P-values refer to Wilcoxon test.

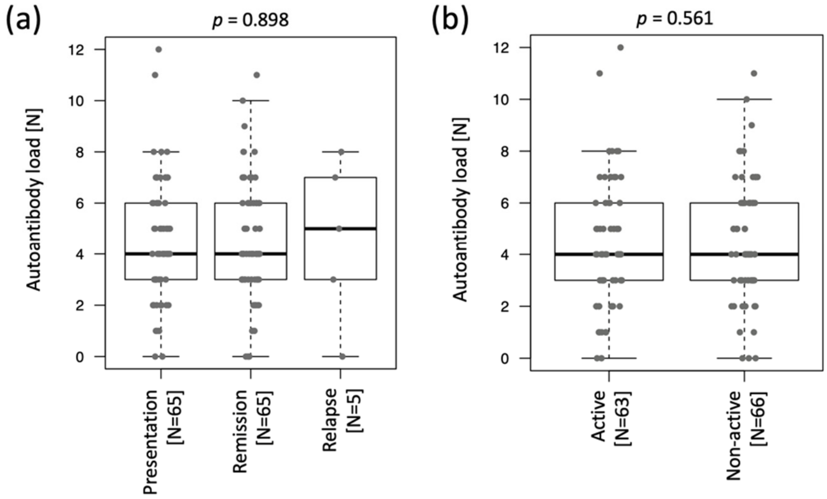

Supplementary Figure S8. Anti-kinesin autoantibody load in AAV longitudinal samples. Autoantibody load in serum samples collected from 65 AAV patients at (a) presentation, remission and relapse and (b) classified as active and non-active. Data on activity status were missing for 6 samples. P-values refer to Kruskal-Wallis test (a) and Wilcoxon test (b).

Supplementary Tables

Supplementary Table S1. AAV cohort: demographics and samples.

**Demographics**	**MUW[a] (n = 41)**	**UCAM[a] (n = 78)**	**UMCG[a] (n = 18)**	**Total patients (n = 137)**
**Sex, n(%)**				
Female	16 (39)	36 (46)	12 (67)	64 (47)
Male	25 (61)	42 (54)	6 (33)	73 (53)
**Age, median (range)[b]**	64 (22-82)	62 (19-85)	56 (41-72)	62 (19-85)
**Diagnosis, n(%)[c]**				
MPA	7 (17)	41 (53)	0 (0)	48 (35)
GPA	7 (17)	37 (47)	18 (100)	62 (45)
**ANCA serology, n(%)**				
Anti-MPO	23 (56)	33 (42)	0 (0)	56 (41)
Anti-PR3	18 (44)	42 (54)	17 ()	77 (56)
Double positive	0 (0)	2 (3)	0 (0)	2 (1)
Negative	0 (0)	1 (1)	1 ()	1 (0.7)
**Organ involvement, n(%)**				
Kidney	12 (29)	43 (55)	-	55 (40)
ENT	6 (14)	22 (28)	-	28 (20)
Respiratory	0 (0)	22 (28)	-	22 (16)
**Treatment at presentation, n(%)[c]**				
Yes	10 (24)	11 (14)	-	21 (15)
No	20 (49)	66 (85)	-	86 (63)
**BVAS at presentation, median (range)[d]**	19 (0-41)	-	-	-
**Timepoint of sample collection, n samples/n patients(% patients)**				
Presentation/Diagnosis	30/30 (73)	78/78 (100)	18/18 (100)	126/126 (92)

Remission	33/22 (54)	33/33 (42)	17/17 (94)	83/72 (53)
Relapse	17/15 (37)	3/3 (4)	0/0 (0)	20/18 (13)

[a]MUV: Medical University of Vienna, Austria; UMCG: University Medical Center, Groningen, Netherlands;
[b]Age at first encounter available for 134 out of 137 AAV patients;
[c]When the total number do not correspond to the total number of individuals it is due to missing data.
[d]Parameter available for 25 samples at diagnosis. One patient was inactive at diagnosis (BVAS=0).

Supplementary Table S2. List of the 151 protein fragments included in the antigen bead array applied in study phase 2.

	Gene	Uniprot ID	Aminoacid position	Selection criteria
1	TRIM21	P19474	200-239	Literature
2	ITGB2	P05107	288-390	Literature
3	FLT1	P17948	619-739	Literature
4	CENPF	P49454	620-761	Literature
5	TMEM100	Q9NV29	1-47	Untargeted screening
6	SLC39A2	Q9NP94	125-159	Literature
7	LMNA	P02545	68-217	Literature
8	AFAP1L1	Q8TED9	525-659	Literature
9	TEK	Q02763	377-510	Literature
10	TEK	Q02763	53-174	Literature
11	KDR	P35968	590-721	Literature
12	ANXA1	P04083	1-102	Literature
13	HSPA4	P34932	699-808	Literature
14	MSN	P26038	359-459	Literature
15	SSB	P05455	15-126	Literature
16	SSB	P05455	156-217	Literature
17	ANGPT2	O15123	215-329	Literature
18	MYCT1	Q8N699	1-25	Literature
19	TNNT2	P45379	4-85	Literature
20	PSMC5	P62195	35-155	Literature
21	LCP1	P13796	18-77	Literature
22	SNRPC	P09234	1-54	Literature
23	TOP1	P11387	93-151	Literature
24	SARDH	Q9UL12	457-561	Literature
25	SARDH	Q9UL12	139-262	Literature
26	CAVIN4	Q5BKX8	276-358	Literature
27	BCL6B	Q8N143	170-248	Literature
28	KRT15	P19012	2-61	Literature
29	KRT15	P19012	398-456	Literature
30	ARHGEF15	O94989	719-813	Literature

31	FH	P07954	425-504	Literature
32	DBT	P11182	36-110	Literature
33	DBT	P11182	131-212	Literature
34	INAVA (C1orf106)	Q3KP66	36-123	Literature
35	AARS2	Q5JTZ9	770-837	Untargeted screening
36	MAP2K6	P52564	2-61	Literature
37	KIF4A	O95239	376-461	Untargeted screening
38	IL6	P05231	178-212	Literature
39	COL4A3	Q01955	112-167	Literature
40	IFNB1	P01574	24-101	Literature
41	SEC24C	P53992	222-306	Literature
42	EPB41L5	Q9HCM4	458-543	Literature
43	KIF15	Q9NS87	671-748	Untargeted screening
44	DNAH12	Q6ZR08	2350-2449	Literature
45	CD34	P28906	154-225	Literature
46	SPON2	Q9BUD6	41-111	Untargeted screening
47	ZFYVE28	Q9HCC9	510-585	Untargeted screening
48	KIAA0232	Q92628	443-537	Untargeted screening
49	AGGF1	Q8N302	429-512	Untargeted screening
50	ANKRD16	Q6P6B7	192-277	Untargeted screening
51	SUV39H2	Q9H5I1	158-226	Untargeted screening
52	CDC42EP2	O14613	54-107	Untargeted screening
53	FEZ1	Q99689	78-156	Untargeted screening
54	H2AX	P16104	118-143	Literature
55	KHSRP	Q92945	188-238	Literature
56	PIK3C2G	O75747	9-100	Literature
57	WHAMM	Q8TF30	439-511	Literature
58	IL12B	P29460	135-225	Literature
59	IL12B	P29460	32-116	Literature
60	HOXD10	P28358	58-144	Untargeted screening
61	ESAM	Q96AP7	132-209	Literature
62	ESAM	Q96AP7	275-332	Literature
63	RBBP6	Q7Z6E9	1546-1640	Literature
64	EHD2	Q9NZN4	388-476	Literature
65	SNRPA	P09012	93-165	Literature
66	H2B2F	Q5QNW6	60-86	Literature
67	H4	P62805	47-90	Literature
68	H3-5	Q6NXT2	96-120	Literature
69	POTEE	Q6S8J3	691-714	Literature
70	USHBP1	Q8N6Y0	121-238	Literature
71	USHBP1	Q8N6Y0	556-641	Literature
72	H1-3	P16402	25-51	Literature

73	ENO3;ENO2;ENO1	P13929;P09104;P06733	98-122	Literature
74	CLEC14A	Q86T13	233-327	Literature
75	CAVIN1 (PTRF)	Q6NZI2	23-98	Literature
76	NPM1	P06748	242-273	Literature
77	GNG11	P61952	20-43	Literature
78	TNF	P01375	1-32	Literature
79	ANXA2	P07355	1-77	Literature
80	FCHSD1	Q86WN1	423-507	Untargeted screening
81	DSC3	Q14574	345-386	Literature
82	F2	P00734	415-521	Literature
83	VCL	P18206	173-321	Literature
84	LEKR1	Q6ZMV7	1-93	Untargeted screening
85	ADCY4	Q8NFM4	491-582	Literature
86	LYZ	P61626	18-72	Literature
87	ITPR2	Q14571	2418-2479	Untargeted screening
88	ACTN4	O43707	554-684	Literature
89	FAM81A	Q8TBF8	253-359	Literature
90	LTF	P02788	363-399	Literature
91	ANGPT2	O15123	176-244	Literature
92	SNAPC2	Q13487	195-330	Untargeted screening
93	FILIP1	Q7Z7B0	978-1058	Literature
94	CAT	P04040	433-519	Literature
95	ROBO4	Q8WZ75	371-456	Literature
96	POLR2L	P62875	33-63	Untargeted screening
97	CEBPA	P49715	56-89	Literature
98	ECSCR	Q19T08	24-71	Literature
99	DLG1	Q12959	420-465	Untargeted screening
100	CAV1	Q03135	4-52	Literature
101	PDZRN3	Q9UPQ7	584-647	Untargeted screening
102	SNRPC	P09234	34-80	Literature
103	HINT2	Q9BX68	33-84	Untargeted screening
104	UBALD1	Q8TB05	81-177	Untargeted screening
105	ZDHHC5	Q9C0B5	622-694	Untargeted screening
106	ZNF618	Q5T7W0	202-300	Literature
107	BCL6B	Q8N143	255-325	Literature
108	CSF2	P04141	109-142	Literature
109	KCNK5	O95279	342-414	Untargeted screening
110	LHFPL6	Q9Y693	142-168	Literature
111	TAGLN3	Q9UI15	19-46	Literature
112	SNRPD1	P62314	1-35	Literature
113	RO60	P10155	3-145	Literature
114	S100A9	P06702	3-100	Literature

115	ANOS1	P23352	63-138	Literature
116	BTF3L4	Q96K17	111-142	Untargeted screening
117	ZNF202	O95125	295-379	Untargeted screening
118	KCNB2	Q92953	507-585	Literature
119	CNGA1	P29973	1-43	Untargeted screening
120	XAGE3	Q8WTP9	74-98	Untargeted screening
121	CABP4	P57796	95-147	Untargeted screening
122	PIGG	Q5H8A4	316-389	Untargeted screening
123	FAM174A	Q8TBP5	79-122	Untargeted screening
124	PRR16	Q569H4	1-60	Literature
125	TOP1	P11387	28-78	Literature
126	CCNI2	Q6ZMN8	9-59	Literature
127	BIRC7	Q96CA5	153-215	Untargeted screening
128	MYBPH	Q13203	19-74	Untargeted screening
129	LIN7A	O14910	44-91	Untargeted screening
130	LDLRAD1	Q5T700	135-174	Untargeted screening
131	IFITM10;AC068580.4	A6NMD0	176-205	Untargeted screening
132	RTEL1	Q9NZ71	253-342	Untargeted screening
133	VCL	P18206	439-536	Literature
134	CDH7	Q9ULB5	666-722	Untargeted screening
135	TPO	P07202	867-932	Literature
136	COX4I2	Q96KJ9	124-152	Literature
137	MNDA	P41218	85-173	Literature
138	DLST	P36957	5-36	Literature
139	MMRN2	Q9H8L6	214-301	Literature
140	SCG2	P13521	251-335	Literature
141	PTX3	P26022	322-381	Literature
142	AZU1	P20160	55-116	Literature
143	ACTN1	P12814	561-631	Literature
144	C5	P01031	322-420	Literature
145	EPAS1	Q99814	538-619	Literature
146	FTMT	Q8N4E7	52-77	Literature
147	SOX15	O60248	179-233	Untargeted screening
148	OSBPL10	Q9BXB5	129-276	Untargeted screening
149	PDIA3	P30101	101-218	Literature
150	MNDA	P41218	176-290	Literature
151	GRN	P28799	128-271	Literature

Supplementary Table S3. Summary of the kinesin superfamily members and number of protein fragments included in the phase 3 of the study.

Gene symbol	Uniprot ID	Protein fragments [N]
KIF1A	Q12756	2
KIF1B	O60333	4
KIF1C	O43896	5
KIF2A	O00139	2
KIF2B	Q8N4N8	3
KIF2C	Q99661	2
KIF3A	Q9Y496	3
KIF3B	O15066	2
KIF3C	O14782	2
KIF4A	O95239	3
KIF5A	Q12840	3
KIF5B	P33176	2
KIF5C	O60282	2
KIF6	Q6ZMV9	2
KIF7	Q2M1P5	1
KIF9	Q9HAQ2	4
KIF11	P52732	2
KIF12	Q96FN5	3
KIF13A	Q9H1H9	2
KIF13B	Q9NQT8	2
KIF14	Q15058	3
KIF15	Q9NS87	3
KIF16B	Q96L93	2
KIF17	Q9P2E2	2
KIF18A	Q8NI77	2
KIF18B	Q86Y91	5
KIF19	Q2TAC6	2
KIF20A	O95235	3
KIF20B	Q96Q89	2
KIF21A	Q7Z4S6	2
KIF21B	O75037	4
KIF22	Q14807	2
KIF23	Q02241	2
KIF24	Q5T7B8	4
KIF25	Q9UIL4	3
KIF26A	Q9ULI4	2
KIF26B	Q2KJY2	4

KIF27	Q86VH2	3
KIFAP3	Q92845	2
KIFC1	Q9BW19	2
KIFC2	Q96AC6	2
KIFC3	Q9BVG8	3
KLC1	Q07866	2
KLC2	Q9H0B6	2
KLC3	Q6P597	2
KLC4	Q9NSK0	2

Supplementary Table S4. List of the 118 protein fragments included in the kinesin screening (study phase 3).

	Gene	Uniprot ID	Aminoacid position
1	KIF1A	Q12756	1389-1512
2	KIF1A	Q12756	906-1020
3	KIF1B	O60333	1633-1704
4	KIF1B	O60333	1036-1122
5	KIF1B	O60333	864-965
6	KIF1B	O60333	369-461
7	KIF1C	O43896	834-891
8	KIF1C	O43896	996-1096
9	KIF1C	O43896	882-978
10	KIF1C	O43896	777-881
11	KIF1C	O43896	354-435
12	KIF2A	O00139	35-144
13	KIF2A	O00139	558-705
14	KIF2B	Q8N4N8	99-182
15	KIF2B	Q8N4N8	520-576
16	KIF2B	Q8N4N8	7-89
17	KIF2C	Q99661	580-698
18	KIF2C	Q99661	3-90
19	KIF3A	Q9Y496	551-697
20	KIF3A	Q9Y496	416-547
21	KIF3A	Q9Y496	431-505
22	KIF3B	O15066	400-450
23	KIF3B	O15066	599-742
24	KIF3C	O14782	639-683
25	KIF3C	O14782	688-761
26	KIF4A	O95239	376-461
27	KIF4A	O95239	477-560

28	KIF4A	O95239	994-1075
29	KIF5A	Q12840	916-1027
30	KIF5A	Q12840	649-785
31	KIF5A	Q12840	535-585
32	KIF5B	P33176	372-433
33	KIF5B	P33176	699-781
34	KIF5C	O60282	743-783
35	KIF5C	O60282	504-579
36	KIF6	Q6ZMV9	17-86
37	KIF6	Q6ZMV9	347-428
38	KIF7	Q2M1P5	1201-1311
39	KIF9	Q9HAQ2	686-771
40	KIF9	Q9HAQ2	369-497
41	KIF9	Q9HAQ2	105-224
42	KIF9	Q9HAQ2	6-96
43	KIF11	P52732	637-784
44	KIF11	P52732	373-477
45	KIF12	Q96FN5	340-422
46	KIF12	Q96FN5	555-646
47	KIF12	Q96FN5	430-513
48	KIF13A	Q9H1H9	879-974
49	KIF13A	Q9H1H9	1595-1682
50	KIF13B	Q9NQT8	1737-1826
51	KIF13B	Q9NQT8	1369-1453
52	KIF14	Q15058	158-230
53	KIF14	Q15058	1088-1165
54	KIF14	Q15058	917-985
55	KIF15	Q9NS87	961-1042
56	KIF15	Q9NS87	765-858
57	KIF15	Q9NS87	671-748
58	KIF16B	Q96L93	1221-1315
59	KIF16B	Q96L93	835-925
60	KIF17	Q9P2E2	935-1028
61	KIF17	Q9P2E2	433-526
62	KIF18A	Q8NI77	536-628
63	KIF18A	Q8NI77	713-802
64	KIF18B	Q86Y91	6-102
65	KIF18B	Q86Y91	719-794
66	KIF18B	Q86Y91	591-694
67	KIF18B	Q86Y91	493-573
68	KIF18B	Q86Y91	418-491
69	KIF19	Q2TAC6	362-470

70	KIF19	Q2TAC6	4-82
71	KIF20A	O95235	774-857
72	KIF20A	O95235	16-105
73	KIF20A	O95235	203-295
74	KIF20B	Q96Q89	916-1063
75	KIF20B	Q96Q89	703-851
76	KIF21A	Q7Z4S6	1057-1128
77	KIF21A	Q7Z4S6	873-942
78	KIF21B	O75037	1202-1274
79	KIF21B	O75037	1123-1199
80	KIF21B	O75037	814-903
81	KIF21B	O75037	494-580
82	KIF22	Q14807	566-648
83	KIF22	Q14807	332-404
84	KIF23	Q02241	802-898
85	KIF23	Q02241	401-545
86	KIF24	Q5T7B8	1160-1242
87	KIF24	Q5T7B8	2-132
88	KIF24	Q5T7B8	773-892
89	KIF24	Q5T7B8	635-719
90	KIF25	Q9UIL4	76-144
91	KIF25	Q9UIL4	276-344
92	KIF25	Q9UIL4	202-270
93	KIF26A	Q9ULI4	253-322
94	KIF26A	Q9ULI4	981-1030
95	KIF26B	Q2KJY2	91-149
96	KIF26B	Q2KJY2	418-507
97	KIF26B	Q2KJY2	1383-1475
98	KIF26B	Q2KJY2	1294-1379
99	KIF27	Q86VH2	1277-1398
100	KIF27	Q86VH2	452-551
101	KIF27	Q86VH2	339-437
102	KIFAP3	Q92845	111-201
103	KIFAP3	Q92845	596-670
104	KIFC1	Q9BW19	249-295
105	KIFC1	Q9BW19	151-218
106	KIFC2	Q96AC6	535-597
107	KIFC2	Q96AC6	200-296
108	KIFC3	Q9BVG8	365-444
109	KIFC3	Q9BVG8	565-676
110	KIFC3	Q9BVG8	445-530
111	KLC1	Q07866	6-80

112	KLC1	Q07866	487-550
113	KLC2	Q9H0B6	1-66
114	KLC2	Q9H0B6	470-534
115	KLC3	Q6P597	1-31
116	KLC3	Q6P597	151-194
117	KLC4	Q9NSK0	1-83
118	KLC4	Q9NSK0	484-570
